# Manifestation of Systemic Toxicity in Rats after a Short-Time Inhalation of Lead Oxide Nanoparticles

**DOI:** 10.3390/ijms21030690

**Published:** 2020-01-21

**Authors:** Marina P. Sutunkova, Svetlana N. Solovyeva, Ivan N. Chernyshov, Svetlana V. Klinova, Vladimir B. Gurvich, Vladimir Ya. Shur, Ekaterina V. Shishkina, Ilya V. Zubarev, Larisa I. Privalova, Boris A. Katsnelson

**Affiliations:** 1 The Medical Research Center for Prophylaxis and Health Protection in Industrial Workers, 30 Popov Str., 620014 Ekaterinburg, Russia; marinasutunkova@yandex.ru (M.P.S.); solovyevasn@ymrc.ru (S.N.S.); chernishov@ymrc.ru (I.N.C.); klinovasv@ymrc.ru (S.V.K.); gurvich@ymrc.ru (V.B.G.); privalovali@yahoo.com (L.I.P.); 2 The Institute of Natural Sciences, the Ural Federal University, 620000 Ekaterinburg, Russia; vladimir.shur@urfu.ru (V.Y.S.); ekaterina.shishkina@urfu.ru (E.V.S.); i.v.zubarev@urfu.ru (I.V.Z.)

**Keywords:** nanoparticles, lead oxide, inhalation exposure, toxicity

## Abstract

Outbred female rats were exposed to inhalation of lead oxide nanoparticle aerosol produced right then and there at a concentration of 1.30 ± 0.10 mg/m^3^ during 5 days for 4 h a day in a nose-only setup. A control group of rats were sham-exposed in parallel under similar conditions. Even this short-time exposure of a relatively low level was associated with nanoparticles retention demonstrable by transmission electron microscopy in the lungs and the olfactory brain. Some impairments were found in the organism’s status in the exposed group, some of which might be considered lead-specific toxicological outcomes (in particular, increase in reticulocytes proportion, in δ-aminolevulinic acid (δ-ALA) urine excretion, and the arterial hypertension’s development).

## 1. Introduction

Lead oxide nanoparticles (PbO-NP) are engineered for some essential technical applications (e.g., magnetic data storage and magnetic resonance imaging). However, from the standpoint of human health risk assessment, it is much more important that PbO particles pollute workplace and ambient air in aerosol form in such long-established and major industries as copper and lead smelters and refineries. These polluting aerosols, resulting from the evaporation of molten metal in pyrometallurgy and its condensation in the air due to cooling and oxidation, contain a substantial proportion of particles falling within the nano-range [1].

Thus, PbO-NPs pose a real inhalation exposure threat, which makes it essential to assess the latter in toxicological experiments. However, we are aware of only one study of this kind published in 2017 by a group of Czech researchers [2].

The study involved an experiment on ICR white mice continuously exposed in a whole-body inhalation setup for 6 weeks to PbO-NP aerosol generated in situ with a mean particle diameter of 25.9 nm (in the size range of 8–230 nm) with an average concentration of 121.7 µ/m^3^. The researchers measured the lead contents of organs and tissues with the visualization of retained nanoparticles and described pathological changes in them, concluding that “subchronic exposure to lead oxide nanoparticles has profound negative effects at both cellular and tissue levels”. Particularly noteworthy are changes in the hippocampus associated with the penetration of nanoparticles into the brain from the nose as a primary site for their deposition. Such penetration is characteristic of inhaled NPs of any chemical composition, as has been discovered in a number of studies [3,4,5], including ours [6,7,8].

However, it is well known that (in contrast to changes in the brain) in a whole-body inhalation chamber, animals’ fur is inevitably soiled with particles, which are then licked off by them; thus, not all toxic effects can be attributed precisely and only to inhalation exposure. It is also regrettable that this study did not consider any of the functional or biochemical indicators of organ and systemic toxicity.

There is no doubt that this toxicity is an inherent feature of PbO-NP at all biological levels—from cellular to systemic. It has been shown, in particular, under subchronic intoxication caused in vivo by repeated intraperitoneal injections of PbO nanosuspension to rats [9] and in vitro by its addition to the incubation medium for human fibroblasts [10]. It would be difficult to think of any a priori causes why the inhalation of PbO-NP should not cause lead poisoning. However, this question is so important for occupational medicine and health risk assessment in the above-mentioned industrial conditions that it must be resolved through experimental studies rather than speculatively.

As the first step in this direction, we are presenting the results of an inhalation experiment in which rats were exposed to PbO-NP aerosol at a concentration around 10 times higher than in the above-mentioned experiment [2]. However, in contrast to the latter, it lasted only five days with daily exposures, which were relatively short because of the restrained positioning of the animals in the “nose only” inhalation setup (see Figure 5). We are convinced, though, that this disadvantage is offset by a very important advantage of such setups—they exclude the possibility of nanoparticles penetrating into the organism by routes other than inhalation and the related uncertainty of the causal relationships between just inhalation exposure at a given level and toxic effects.

Overall, the inhalation period in our experiment was 50 times shorter compared with the experiment [2], and the total exposure to nanoparticles may be estimated as about five times lower, even though the concentration of nanoparticles was 10 times higher.

The purpose of this paper is to demonstrate that signs of intoxication were detectable even under an exposure as low and short as in our experiment.

## 2. Results and Discussion

Previously, we demonstrated that the deposition of nanoparticles in the lungs as a result of intratracheal administration [11,12,13,14] or inhalation exposure [6,7,8] caused a response in the lower airways that was essentially similar to but a lot more intensive than the one provoked by similar exposures to low-soluble highly cytotoxic micrometer particles, such as, for instance, quartz dust [15,16]. This response manifests itself as an increase in the cellularity of the bronchoalveolar lavage fluid (BALF), mainly at the expense of neutrophil leukocytes. The increase in proteins, in some enzymes (particularly of lysosomal or partly lysosomal origin) and in a number of other biochemical components of the BALF extracellular fraction also testifies for inhaled PbO-NP’s pulmonary toxicity.

As follows from the results presented in Table 1 and Table 2, we observed the same in our experiment. Although the differences from the corresponding indices of the control group’s BALF appear to be rarely statistically significant, the mutual correspondence of the resulting changes allows us to consider them to be an actual effect of inhalation exposure to nanoparticles. 

As was stated in the Introduction, neither the total exposure to lead nor the period for related adverse changes to develop in the organism might be estimated as considerable, which reduced the probability of development of lead poisoning. Indeed, many of the indices listed in Table 3 indicate that it was of low to moderate intensity.

Note, first of all, that body mass reduction, which was observed in the sham-exposed group as well, was not enhanced by lead exposure. This reduction was likely to be an immediate consequence of the subacute stress caused by immobilization at the beginning of the experiment when the rats were not yet sufficiently well adapted to it. Incidentally, we did not observe this effect during months of exposure in the same setup in three different experiments involving other NP species [6,7,8].

The masses of the internal organs (both absolute and related to body mass) did not differ statistically significantly from the control values. However, still, these indices were noticeably, even if not significant statistically, increased for lungs, the first target organ for inhaled nanoparticles; for kidneys, the main organ eliminating lead from the blood, which is well-known to suffer damage under lead intoxication (e.g., [17,18,19]); and for heart, whose electrocardiogram (ECG) signs of impairment will be described below.

The shift in the balance between the processes of excitation and inhibition in the central nervous system (CNS) toward inhibition is evidenced by a statistically significant increase in the index of temporal summation of sub-threshold impulses. Although none of the three behavior indices that we recorded was statistically significantly different from the control values, still, the unidirectional increase in these indices (number of head-dips into holes, number of crossed squares, total number of movements on the “open field”) suggests enhanced exploratory activity.

The impairment of the porphyrin metabolism, which is specific to lead toxicodynamics, manifested itself in a more than twofold and statistically significant increase in the concentration of δ-aminolevulinic acid (δ-ALA) in urine, which is one of the early signs of lead poisoning. Typical of it is also an increased concentration of coproporphyrin in urine, which in this study was noticeable, even if statistically not significant. As is known, such impairment is a prerequisite to hem synthesis suppression; however, in this experiment, it did not reach a clearly detectable level, since neither the whole blood nor erythrocyte hemoglobin content was decreased at all. Lead anemia did not develop contrary to expectations judging also by no reduction in the erythrocyte count; however, already there was an appreciable and statistically significant compensatory enhancement of erythropoiesis which manifested itself as a considerable increase in the proportion of reticulocytes, another one of the most sensitive effects of lead intoxication.

It should be noted also that the number of micronuclei in the polychromatophilic erythrocytes of the bone marrow was more than doubled. Although this shift was not statistically significant, it is noteworthy since we practically always discover a systemic genotoxic effect under exposure to various metal oxide nanoparticles judging by increased DNA fragmentation coefficient, which is another informative genotoxicity index. For instance, it was significantly increased under subchronic intoxication caused by repeated intraperitoneal injections of PbO-NPs [9].

Turning back to the hematological features of lead intoxication, only some of the white blood indices revealed a noticeable, although statistically insignificant increase. These shifts are also noteworthy because they are fairly typical of experimental lead intoxications and, in some studies, were even more substantial than in the present one and statistically significant at that (e.g., [20,21,22,23,24]).

Proceeding now to the blood serum, note that the total protein and protein fractions in it were not different from the control value. At the same time, some biochemical indices point to damage to hepatocytes, which, as is well known, may show itself not only as enzyme biosynthesis inhibition (which manifested itself as a statistically significant decrease in the serum activity of γ-glutamyl transpeptidase and an insignificant decrease in the activity of alkaline phosphatase) but also as enhanced enzyme release (which led to an increased serum activity of aminotransferases, lactate dehydrogenase, and amilase). As can be seen in Table 3, some of these shifts are statistically significant.

The small increase in the kidney mass, although statistically insignificant, may assumingly be associated with nephrotoxicity, which is lead’s other highly characteristic feature [17,18,19]. We can interpret in the same way both the increased protein content of the urine and the statistically significant, albeit small decrease in urine’s specific density (despite a somewhat lower volume of diuresis) and significantly increased creatinine content (while the creatinine content of the blood serum was not changed). The δ-ALA content of the urine was increased statistically significantly and much higher than in the blood serum. Note that such combination of signs of damage to both tubular epithelium and Malpighian glomeruli (confirmed morphologically) had been observed under subchronic intraperitoneal exposure to PbO-NP [9].

The indices of calcium, myoglobin, troponin, natriuretic peptide, endothelin-1, and vascular endothelial growth factor (VEGF) content of the blood serum were studied as markers of a possible vasocardiotoxic effect of lead intoxication discussed below, but none of them revealed any substantial shift.

The data in Table 4 provide evidence that the lead exposure caused interrelated, although statistically insignificant, hemodynamic changes in the rat tail measured post exposure. All the three blood pressure levels (systolic, diastolic, and mean) were elevated compared with the control values. The natural assumption that this arterial hypertension was caused by increased systemic resistance to blood flow is in agreement with the decelerated blood flow and decreased blood volume. It is important to note that we had also discovered that all these shifts under subchronic intoxication were caused by intraperitoneal injections of lead acetate [24] but, to the best of our knowledge, nobody had previously estimated hemodynamic parameters under PbO-NP exposure of any kind.

It should be noted that many of the epidemiological studies have provided evidence of a cause–response relationship between human exposure to lead and the prevalence of arterial hypertension [25,26,27,28,29]. Animal experiments have also been performed, seeking mainly to identify the possible mechanisms of lead-induced hypertension [30,31,32,33,34]. However, the author of a relatively recent overview [35] concluded that “in an occupational setting, the effect of lead exposure on blood pressure remains controversial”. In this context, it is to be recalled that the air inhaled by metallurgy workers is contaminated with lead in aerosol form, containing a considerable fraction of nanoparticles (see the Introduction). Therefore, the evidence obtained by us for the first time ever that even a very moderate intoxication caused by exposure to PbO nanoaerosol provokes shifts of a hypertensive type cannot be ignored.

Table 5 presents ECG analysis results obtained in two standard leads, and it is worthwhile to compare them with the data of our ECG study under subchronic lead acetate intoxication [24]. Whereas in the latter we observed elongation of the majority of the interwave intervals pointing to the slowing of the heart rate, in the current experiment, changes in the ECG intervals in the absence of bradycardia were of different types, with the only shift that was statistically significant being of opposite sign (QRS shortening). The statistically significant increase of the P and T amplitudes discovered in both ECG leads in this experiment was not observed in the previous experiment with lead acetate. At the same time, both studies had a common feature, a lowered isoelectric ECG line in the second lead, which can point to some impairment of the myocardium or, at least, metabolic disturbances in it. Such disturbances were indeed revealed in the hearts of rats suffering subchronic lead intoxication [24].

**Figure 1:**
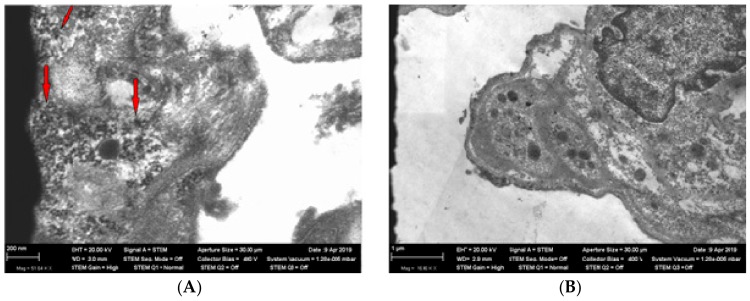
Panel (**A**)—nanoparticles (shown by arrows) in alveolocytes of type 1 (TEM, magnification ×51,640) in rat lungs from the exposed group. Panel (**B**)—alveolocytes of type 1 (TEM, magnification ×16,480) in rat lungs from the control (sham-exposed) group.

**Figure 2:**
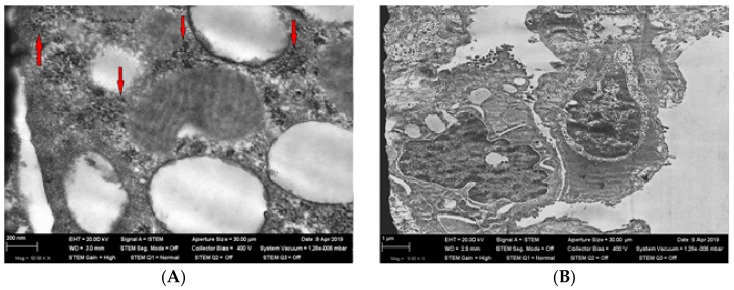
Panel (**A**)—nanoparticles (shown by red arrows) in alveolocytes of type 2 (TEM, magnification ×50,580) in rat lungs from the exposed group. Panel (**B**)—alveolocytes of type 2 (TEM, magnification ×9000) in rat lungs from the control (sham-exposed) group.

Transmission electron microscopy of the lung tissue revealed nanoparticles in the cytoplasm of type 1 and type 2 alveolocytes (Figure 1 and Figure 2).

**Figure 3:**
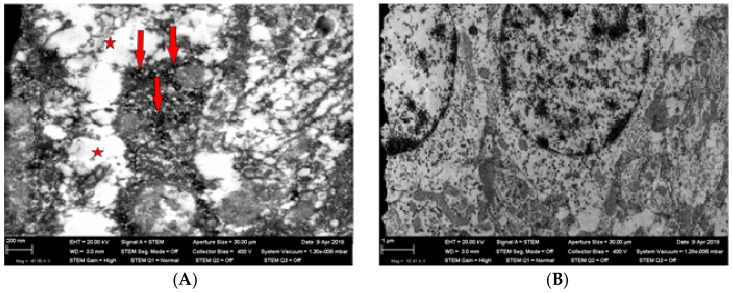
Panel (**A**)—a neuron body with numerous nanoparticles (shown by red arrows) and vacuolized cytoplasm (shown by a red asterisk); TEM, magnification ×41,950; Panel (**B**)—non-damaged neuron bodies in the olfactory region of a rat brain from the control (sham-exposed) group; TEM, magnification ×10,410.

**Figure 4:**
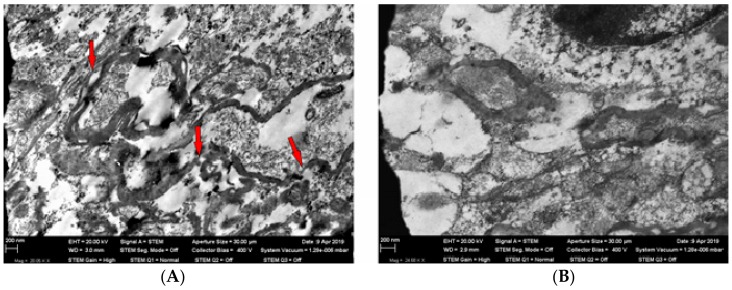
Panel (**A**)—an axon with signs of membrane demyelinization (shown by red arrows) in the olfactory region of a rat brain from the exposed group; TEM, magnification ×20,050. Panel (**B**)—non-damaged axon myelin sheath in the olfactory region of a rat brain from the control (sham-exposed) group; TEM, magnification ×24,680.

Electron microscopy of the olfactory region of the brain showed cytoplasmic vacuolization of neurons, numerous nanoparticles in the neurons’ bodies (Figure 3), and pronounced demyelinization of axon membranes (Figure 4).

The mechanism underlying the penetration of inhaled nanoparticles into the brain neurons was mentioned in the Introduction. We should just emphasize that such penetration is also associated with some ultra-structural changes in the brain tissue. It can be assumed that the somewhat controversial shifts in the CNS functional indices that we discussed above are connected not only and even not so much with systemic lead intoxication as with a direct effect of cytotoxic nanoparticles on the brain structures. In this connection, we should recall the key role of the sense of smell in animal behavior control.

## 3. Materials and Methods

**Figure 5:**
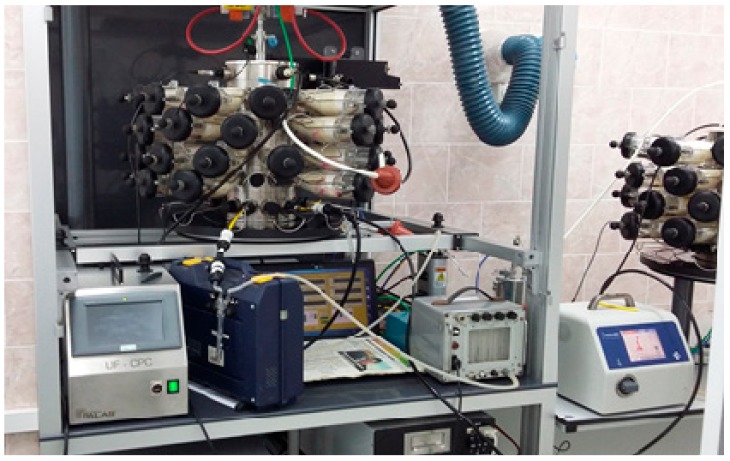
The nose-only inhalation setup (photographed without the door wings of the draught cupboard) and a similar tower for the sham exposure of the control group.

Airborne Pb-NPs were obtained by sparking from 99.9999% pure lead rods with a diameter of 5.6 mm (supplied by “Giredmet Ltd.”—Moscow, Russia) using a Palas DNP-3000 generator set at “Medium energy” regime with the current strength of 5 A and nitrogen flow of 8 L/min. Then, this flow was being mixed with air (6 L/min) for cooling and for oxidizing Pb into PbO-NPs, which were fed into a nose-only exposure tower (CH Technologies, Westwood, NJ, USA) with rats placed into individual restrainers (Figure 5).

**Figure 6:**
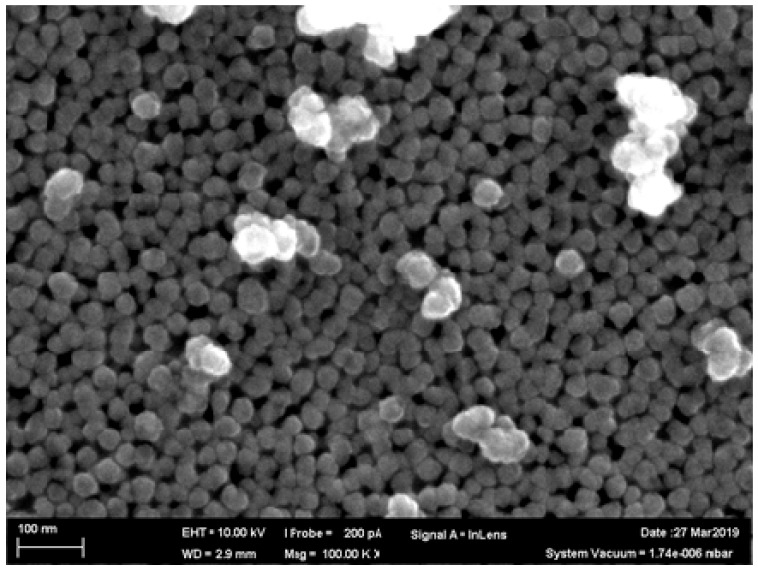
Nanoparticles retained by the Whatman Anodisc membrane filter (mesh diameter 20 nm) of the inhalation setup. SEM, magnification ×100,000.

**Figure 7:**
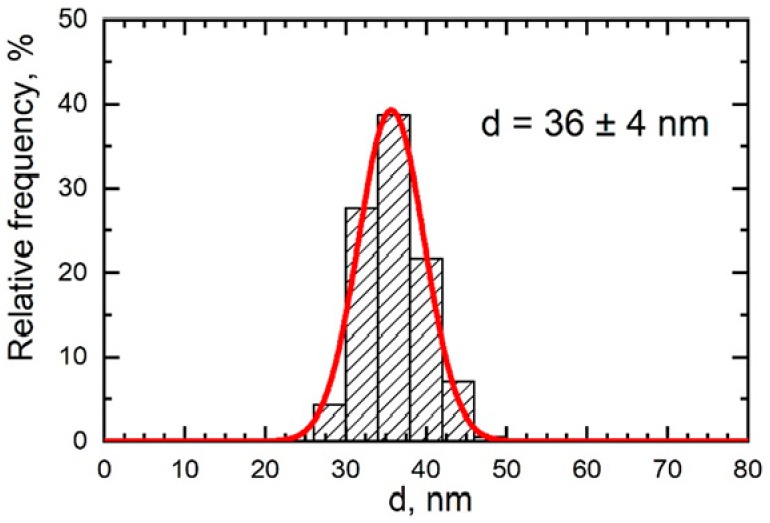
Particle or particle aggregate size normal distribution function (red curve) obtained by statistical processing of 360 measured SEM images of particles accumulated on a polycarbonate filter from the exposed rats’ breathing zone.

A setup of the same design obtained from the same supplier was used for a sham exposure of control rats. Particles collected on filters and inspected under a scanning electron microscope (SEM) had a spherical shape and either were singlets or formed small aggregates (Figure 6). The latter, if compact, were measured as one particle. Even so, the particle size distribution (Figure 7) proved fairly clean-cut and restricted to the nanometric range with a mean (± s.d.) diameter of 36 ± 4 nm. The chemical composition of particles sampled on the filters was confirmed by Raman spectroscopy to be PbO (the obtained spectrum had two characteristic peaks at wavelengths 82 cm^−1^ and 147 cm^−1^ which, according to [36] correspond to PbO).

Our experiment was carried out on outbred white female rats from our own breeding colony with the initial body weight of 252 ± 1.5 g in the NP-exposed 14 rats and 251 ± 2.2 g in the sham-exposed 14 rats. All these rats were housed in conventional conditions, breathed unfiltered air, and were fed standard balanced food. The experiments were planned and implemented in accordance with the “International guiding principles for biomedical research involving animals” developed by the Council for International Organizations of Medical Sciences (1985) and were approved by the Ethics Committee of the Ekaterinburg Medical Research Center for Prophylaxis and Health Protection in Industrial Workers.

After a preliminary training, the rats were exposed or sham-exposed for 4 h a day, 5 times during one working week. Along with each single exposure, a sample of airborne nanoparticles was collected on an acetyl cellulose fine fiber filter attached to the inhalation setup instead of a rat’s nose while monitoring the volume velocity of air drawn through the filter. Each daily filter was being sampled during 4 hr in parallel with exposure of rats. The mass of Pb retained on it was determined with an atomic absorption spectrometer, ContrAA 700 (Analytic Jena A, Jena, Germany), and translated into the mass of PbO and then into its air concentration as mg/m^3^, which proved to be equal to 1.30 ± 0.10 mg/m^3^ (The OSHA Permissible Exposure Limit for Pb as well as the respective Russian national standard is 50 µg/m^3^ averaged over an 8-h period which corresponds to 54 µg PbO/m^3^. For the 4 h exposure, the equivalent level would be 108 µg/m^3^. We chose a one order of magnitude higher level to presumably ensure some toxicity outcomes, a short total exposure period notwithstanding).

The mass deposition of inhaled particles in the lower airways can be estimated only very roughly because this estimation is based on physiological parameters that are rather uncertain:

(1) The rat’s minute respiratory ventilation as assessed experimentally by different authors varies between 78 mL [37] and 210 mL [38].
  The so-called multi-path particle dosimetry (MPPD) model for rats used in [39] assumes the breathing frequency to be equal to 102 min^−1^ and tidal volume to be 2.1 mL. It gives minute ventilation, 214 mL, which almost exactly corresponds to the above given highest experimental value and thus seems to be an extreme estimate. In our previous inhalation studies with iron oxide [6], silica [40], and nickel oxide [41] nanoparticles, we calculated particle deposition based on the minute ventilation value of 100 mL, which is close to the median of the above experimental range but of course somewhat arbitrary.
(2) The pulmonary deposition fraction of inhaled NPs may be estimated to be equal to 0.52, which is close to the lowest of the differently substantiated assessments given by [42] but much higher as compared with 0.124, which value is assumed to be the alveolar region deposition fraction for 20 nm particles according to the same MPPD model.

Thus, the pulmonary PbO-NP deposition per each single exposure may be tentatively estimated as mean NP concentration in the inhaled air, mg/m^3^ × minute respiratory ventilation, mL × exposure time, min × mL to m^3^ ratio × mcg to mg ratio × deposited fraction = 1.30 × 100 × 240 × 10^−6^ × 10^3^ × 0.52 = 16.2 mcg.

After the end of the exposure week, the following procedures were performed for all rats:

(1) weighing of the body;
(2) estimation of the CNS ability to evoke temporal summation of sub-threshold impulses (a variant of the withdrawal reflex and its facilitation by repeated electrical stimulations in an intact, conscious rat) [9,19,43,44,45];
(3) recording of the number of head-dips into the holes of a hole-board (which is a simple but informative index of exploratory activity frequently used for studying the behavioral effects of toxicants and drugs) (e.g., [9,19,44,45,46,47]);
(4) collection of daily urine (from rats put into metabolic cages) for analysis of protein, urine specific gravity, pH, urea, uric acid, total coproporphyrin, and δ-aminolevulinic acid (δ-ALA);
(5) bronchoalveolar lavage to obtain fluid (BALF) for cytological and biochemical characterization.

Bronchoalveolar lavage was carried out 24 h after the last inhalation exposure. A cannula connected to a Luer’s syringe containing 10 mL of normal saline was inserted into the surgically prepared trachea of a rat under hexenal anaesthesia. The fluid entered the lungs slowly under the gravity of the piston, with the animal and syringe positioned vertically. Then, the rat and the syringe were turned 180°, and the fluid flowed back into the syringe. The extracted BALF was poured into siliconized refrigerated tubes. An aliquot sample of the BALF was drawn into a white blood cells (WBC) count pipette together with 3% acetic acid and methylene blue. Cell count was performed in a standard hemocytometer (the so-called Goryayev’s Chamber). For cytological examination, the BALF was centrifuged for 4 min at 1000 rpm; then, the fluid was decanted, and the sediment was used for preparing smears on two microscope slides. After air drying, the smears were fixed with methyl alcohol and stained with azure eosin. The smears were microscoped with immersion at a magnification of ×1000. The differential count for determining the percentage of alveolar macrophages (AM), neutrophil leucocytes (NL), and other cells counted together was conducted up to a total number of 100 cells. Allowing for the number of cells in the BALF, these percentages were recalculated in terms of absolute AM and NL counts.

Seven out of 14 not rats not subjected to that lavage in both exposed and sham-exposed groups were killed by cervical dislocation under ether anesthesia. The liver, spleen, kidneys, heart, lungs, and brain were weighed. Blood was collected from the tail vein under ether anesthesia at the end of the experiment. The biochemical indices determined in the blood included total serum protein, albumin, globulin, alanine and aspartate transaminases (ALT, AST), bilirubin, catalase, glutathione, ceruloplasmin, malondialdehyde (MDA), lactate dehydrogenase (LDH), alkaline phosphatase, amilase, glucose, thiol groups (SH-groups), gamma-glutamyl transpeptidase (GGTP), urea, acid, calcium, myoglobin, troponin, natriuretic peptide, endothelin-1, and vascular endothelial growth factor (VEGF).

For determining the hemoglobin content, hematocrit, thrombocrit, mean erythrocyte volume, and for counting a red blood cells (RBC), WBC, and thrombocytes, we used a MYTHIC-18 auto-hematology analyzer (C2 Diagnostic, Montperllier, France). Reticulocyte percentage was counted on smears under optical microscopy after supravital staining with brilliant cresyl blue. The number of micronucleus in the polychromatophilic erythrocytes of the bone marrow was counted in smears under optical microscopy after fixing in methanol and staining by Pappenheim’s stain.

All the clinical laboratory blood and urine tests with the exception of the specially considered ones were performed using the well-known techniques described in many manuals (e.g., [48]). Before performing euthanasia, we measured the heart rate, arterial pressure, blood flow rate, and blood volume in the rat tails using the noninvasive blood pressure system CODA-HT8 (Kent Scientific, Torrington, CT, USA) and recorded electrocardiograms (ECG) in the first and second leads with programmed analysis using the ecgTUNNEL system (emka TECHNOLOGIES, Paris, France).

In addition, the pulmonary and brain accumulation of NPs and the ultrastructure of respective tissues were visualized by means of transmission electron microscopy (TEM). To this end, pieces of an organ were fixed in 2% paraformaldehyde and 2.5% glutaraldehyde in a cacodylate buffer with 5% sucrose at pH 7.3, post-fixed in 1% osmium tetroxide, contrasted with uranyl acetate en bloc, and embedded in epoxy resin (Spurr). This sample preparation procedure was carried out in a microwave tissue processor, HISTOS REM (Milestone, Milan, Italy). Semi-thin (900 nm thick) sections of epoxy blocks were stained in toluidine blue with the addition of 1% borax and examined under the optical microscope for choosing a site for TEM. The 60 nm ultrathin sections of this site obtained with the help an ultramicrotome (Power Tome, RMC, Tucson, AZ, USA) were contrasted with uranyl acetate and lead citrate. Grid-mounted sections were investigated in an electron microscope, AURIGA (Carl Zeiss; MT, Oberkochen, Germany) in the STEM mode in the range of magnifications 1200–200,006.

## 4. Conclusions

The experimental results and their discussion with reference to the literature data enable us to argue that inhalation exposure to lead oxide nanoparticles associated with their retention in the organism (olfactory brain included) demonstrated with TEM, led (even though the exposure level was relatively low and was of short duration) to disturbances in the organism, some of which are specific to lead intoxication (in particular, increase in reticulocytes proportion, in δ-ALA urine excretion, and signs of arterial hypertension).

## Figures and Tables

**Table 1:** Cell counts in the bronchoalveolar lavage fluid (BALF) in rats after repeated inhalation of lead oxide nanoparticles (x ± s.e.). PbO-NP: lead oxide nanoparticles.

Group and Number of Animals	Number of Cells ×10^6^		
	Total	Neutrophil Leukocytes	Alveolar Macrophages
Control (sham-exposed), 7 rats	3.29 ± 0.67	0.35 ± 0.13	2.94 ± 0.64
Exposed to PbO-NP Aerosol, 7 rats	5.29 ± 1.33	0.62 ± 0.12	4.66 ± 1.24

**Table 2:** Some biochemical indices in bronchoalveolar lavage fluid (BALF) supernatant in rats after repeated inhalation of lead oxide nanoparticles (x ± s.e.).

Indices	Group and Number of Animals	
	Control (Sham-Exposure), 7 Rats	Exposed to PbO-NP Aerosol, 7 Rats
Total protein, mg/L	81.68 ± 6.19	133.38 ± 31.52
Alanine aminoransferase (ALT) U/L	5.52 ± 0.80	7.64 ± 0.58
Aspartate aminotransferase (AST), U/L	1.96 ± 0.16	1.42 ± 0.21
De-Ritis coefficient (AST/ALT ratio)	2.77 ± 0.31	5.92 ± 1.00 *
Alkaline phosphatase, U/L	13.62 ± 2.85	45.16 ± 5.17 *
Gamma-glutamyl transpeptidase, U/L	0.46 ± 0.16	7.26 ± 1.07 *
Lactate dehydrogenase, U/L	29.50 ± 5.72	38.20 ± 5.64
Urea, mmol/L	0.22 ± 0.04	0.24 ± 0.07
Glucose, mmol/L	0.00 ± 0.00	0.02 ± 0.02

*—values statistically significantly different from the corresponding values of the control group (*p* ≤ 0.05 by Student’s *t*-test).

**Table 3:** Body and inner organs mass and some functional indices in rats after repeated inhalation of lead oxide nanoparticles (x ± s.e.).

Indices	Group and Number of Animals	
	Control (Sham-Exposure), 7 Rats	Exposed to PbO-NP Aerosol, 7 Rats
**Body and organs mass indices**		
Initial body mass, g	251.43 ± 2.19	251.79 ± 1.54
Final body mass, g	247.50 ± 2.71	250.71 ± 2.82
Body mass gain, %	−1.56 ± 0.70	−0.43 ± 0.92
Heart mass, g	0.77 ± 0.03	0.83 ± 0.03
Lung mass, g	1.67 ± 0.09	1.89 ± 0.10
Liver mass, g	9.03 ± 0.46	9.09 ± 0.42
Kidney mass, g	1.67 ± 0.05	1.75 ± 0.06
Spleen mass, g	0.54 ± 0.02	0.56 ± 0.02
Brain mass, g	1.96 ± 0.04	1.90 ± 0.04
Heart mass, g/100 g of body mass	0.31 ± 0.01	0.33 ± 0.01
Lung mass, g/100 g of body mass	0.68 ± 0.04	0.75 ± 0.03
Liver mass, g/100 g of body mass	3.65 ± 0.16	3.61 ± 0.12
Kidney mass, g/100 g of body mass	0.68 ± 0.02	0.70 ± 0.02
Spleen mass, g/100 g of body mass	0.22 ± 0.01	0.22 ± 0.01
Brain mass, g/100 g of body mass	0.79 ± 0.01	0.76 ± 0.03
**Neurobehavioral tests**		
Number of head-dips into holes during 3 min	13.86 ± 1.46	15.07 ± 1.51
Number of squares crossed during 3 min	31.64 ± 2.17	32.21 ± 2.27
Total number of movements on the “open field” during 3 min	51.21 ± 3.90	56.36 ± 4.54
Temporal summation of sub-threshold impulses, sec	10.24 ± 0.29	13.11 ± 0.20 *
**Hematological indices**		
Hemoglobin, g/L	140.33 ± 2.16	142.57 ± 2.13
Hematocrit, %	21.05 ± 0.27	21.54 ± 0.41
Erythrocytes, 10^12^ cells/L	6.83 ± 0.17	6.86 ± 0.23
Mean corpuscular volume, μm^3^	61.77 ± 0.76	62.99 ± 1.34
Mean corpuscular hemoglobin, 10^−12^ g	20.60 ± 0.24	20.86 ± 0.48
Mean corpuscular hemoglobin concentration, g/L	333.33 ± 2.50	331.14 ± 1.68
Red cell distribution width, %	12.50 ± 0.32	13.40 ± 0.38
Reticulocytes, ‰	26.44 ± 4.57	71.80 ± 7.87 *
Number of micronuclei in the polychromatophilic erythrocytes of bone marrow, ‰	0.25 ± 0.25	0.40 ± 0.24
Thrombocytes, 10^6^ cells/mL	705.00 ± 63.71	813.71 ± 42.92
Thrombocrit, %	0.20 ± 0.01	0.22 ± 0.01
Mean platelet volume, μm^3^	5.27 ± 0.13	5.33 ± 0.10
Leukocytes, 10^6^/mL	6.40 ± 0.34	6.94 ± 0.52
Banded neutrophils, 10^6^/mL	0.07 ± 0.01	0.08 ± 0.01
Segmented neutrophils, 10^6^/mL	1.48 ± 0.12	1.68 ± 0.19
Basophils, 10^6^/mL	0.00 ± 0.00	0.00 ± 0.00
Eosinophils, 10^6^/mL	0.37 ± 0.12	0.28 ± 0.07
Lymphocytes, 10^6^/mL	4.12 ± 0.27	4.45 ± 0.28
Monocytes, 10^6^/mL	0.37 ± 0.03	
**Serum biomarkers**		
Total protein content of blood serum, g/L	81.33 ± 1.21	81.50 ± 1.05
Albumin content of blood serum, g/L	49.56 ± 0.75	50.94 ± 0.95
Globulins of blood serum, g/L	31.77 ± 1.05	30.56 ± 0.62
A/G index	1.57 ± 0.05	1.67 ± 0.05
Alanine aminoransferase (ALT) activity in blood serum, mM/h×L	54.07 ± 2.62	56.91 ± 2.55
Aspartate aminotransferase (AST) activity in blood serum, mM/h×L	226.70 ± 10.22	265.60 ± 14.03 *
de Ritis coefficient (AST/ALT ratio)	4.22 ± 0.20	4.72 ± 0.31
Total bilirubin in blood serum, μmol/L	1.46 ± 0.14	1.21 ± 0.11
Glucose in blood serum, mmol/L	0.39 ± 0.22	0.54 ± 0.16
Thiol groups (SH-groups), mmol/L	0.37 ± 0.02	0.37 ± 0.02
Gamma-glutamyl transpeptidase (GGTP) in blood serum, U/L	4.83 ± 0.25	3.97 ± 0.19 *
Urea in blood serum, mmol/L	7.27 ± 0.49	6.91 ± 0.45
Uric acid in blood serum, μmol/L	92.57 ± 5.97	91.57 ± 6.52
Alkaline phosphatase in blood serum, nmol/(s×L)	198.80 ± 23.43	190.00 ± 20.32
Catalase in blood serum, μmol/L	0.67 ± 0.01	0.65 ± 0.01
Reduced glutathione in the blood hemolysate, μmol/L	35.33 ± 3.11	33.41 ± 3.47
Ceruloplasmin in blood serum, mg/%	123.03 ± 7.22	127.58 ± 6.06
Malonyl dialdehyde (MDA) in blood serum, μmol/L	3.71 ± 0.14	3.98 ± 0.18
Lactate dehydrogenase (LDH), U/L	2590.00 ± 177.21	3171.43 ± 129.31 *
Amilase in blood serum, U/L	3443.71 ± 218.91	4148.83 ± 362.11
Total Ca in blood serum, mmol/L	2.75 ± 0.02	2.75 ± 0.03
Endothelin-1, pg/mL	26.11 ± 2.72	25.62 ± 1.15
Myoglobin, ng/mL	67.35 ± 9.02	35.51 ± 16.39
Troponin, ng/mL	0.059 ± 0.052	0.105 ± 0.103
Natriuretic peptide, pg/mL	1.09 ± 0.08	1.24 ± 0.15
Vascular endothelial growth factor (VEGF), 10^6^ U/mL	3.64 ± 0.69	5.51 ± 2.29
**Urinary biomarkers**		
Diuresis, mL	40.36 ± 2.29	38.17 ± 2.77
Urine specific gravity, g/mL	1.012 ± 0.001	1.010 ± 0.000 *
Urine pH	6.91 ± 0.06	6.63 ± 0.11 *
Protein in urine, g/L	104.43 ± 5.31	123.63 ± 12.36
Total coproporphyrin in urine, μmol	71.28 ± 27.58	120.86 ± 49.59
δ-aminolevulinic acid (ALA) in urine, μg/mL	5.65 ± 0.86	13.97 ± 1.25 *
Urea in urine, mmol/L	115.19 ± 6.63	132.53 ± 7.37
Uric acid in urine, μmol/L	1.55 ± 0.88	9.17 ± 4.93
Creatinine in blood serum, μmol/L	58.60 ± 1.84	56.87 ± 2.98
Creatinine in urine, μmol/L	0.71 ± 0.03	0.93 ± 0.10 *
Endogenous creatinine clearance	0.50 ± 0.04	0.59 ± 0.04

*—values statistically significantly different from the corresponding values of the control group (*p* ≤ 0.05 by Student’s *t*-test).

**Table 4:** Some hemodynamic indices of rats after repeated inhalation of lead oxide nanoparticles (x ± s.e.).

Groups and Number of Animal	Control (Sham-Exposure), 7 Rats	Exposed to PbO-NP Aerosol, 7 Rats
Systolic blood pressure, mmHg	128.32 ± 3.38	135.26 ± 6.09
Diastolic blood pressure, mmHg	91.20 ± 2.63	98.31 ± 4.86
Mean blood pressure, mmHg	103.19 ± 2.86	110.25 ± 5.22
Heart rate, bpm	335.77 ± 7.15	338.85 ± 7.80
Blood flow in the tail, μL/min	32.70 ± 3.07	29.27 ± 1.84
Tail blood volume, μL	122.90 ± 12.12	108.60 ± 7.97

**Table 5:** Electrocardiogram (ECG) readings in rats after repeated inhalation of lead oxide nanoparticles (x ± s.e.).

Indices	Control (Sham-Exposure), 7 Rats,		Exposed to PbO-NP Aerosol, 7 Rats	
	ECG in the 1st Lead	ECG in the 2nd Lead	ECG in the 1st Lead	ECG in the 2nd Lead
Intervals, msec				
RR	161.25 ± 3.75	166.22 ± 3.54	167.27 ± 3.82	162.31 ± 3.71
PQ	45.33 ± 1.56	45.23 ± 1.01	45.20 ± 1.07	46.70 ± 1.10
QRS	33.31 ± 3.02	22.83 ± 0.88	25.58 ± 0.80 *	21.75 ± 0.62
QT	61.12 ± 1.70	70.88 ± 1.70	65.48 ± 2.38	72.56 ± 1.70
QT corrected using Basett’s formula.	152.49 ± 2.86	174.46 ± 3.72	161.29 ± 6.82	180.63 ± 4.30
QT corrected using Friderica’s formula	112.41 ± 2.41	129.18 ± 2.79	119.36 ± 4.77	133.26 ± 3.08
P duration	15.55 ± 0.29	15.89 ± 0.76	15.98 ± 0.41	17.19 ± 1.33
Amplitudes, mV				
Isoelectric line	−0.02 ± 0.00	−0.06 ± 0.00	−0.03 ± 0.00	−0.08 ± 0.00 *
P	0.03 ± 0.00	0.09 ± 0.01	0.05 ± 0.01 *	0.11 ± 0.01 *
Q	−0.0005 ± 0.0006	−0.0008 ± 0.0006	−0.0003 ± 0.0004	−0.0035 ± 0.0016
R	0.27 ± 0.06	0.44 ± 0.03	0.28 ± 0.05	0.50 ± 0.06
S	−0.05 ± 0.02	−0.05 ± 0.03	−0.03 ± 0.01	−0.09 ± 0.03
QRS	0.22 ± 0.06	0.39 ± 0.05	0.25 ± 0.05	0.41 ± 0.06
T	0.03 ± 0.01	0.14 ± 0.02	0.08 ± 0.01 *	0.21 ± 0.02 *

*—values statistically significantly different from the corresponding values of the control group (*p* ≤ 0.05 by Student’s *t*-test).
